# Comparative evaluation of three PCR base diagnostic assays for the detection of pathogenic trypanosomes in cattle blood

**DOI:** 10.1186/1756-3305-1-46

**Published:** 2008-12-24

**Authors:** Samuel M Thumbi, Francis A McOdimba, Reuben O Mosi, Joseph O Jung'a

**Affiliations:** 1 Department of Animal Production, Faculty of Veterinary Medicine, University of Nairobi, Kenya, P.O Box 29053 Nairobi, Kenya; 2 International Livestock Research Institute, P.O Box 30409 Nairobi, Kenya; 3 Institute of Primate Research, P.O Box 24481, 00502 Nairobi, Kenya

## Abstract

Currently, several PCR based diagnostic assays have been developed to improve the detection of pathogenic trypanosomes. These tests include use of species specific primers, single and nested PCRs' based on primers amplifying the Internal Transcribed Spacer (ITS) regions of ribosomal DNA. This study compares three PCR based diagnostic assays and assesses the agreement of these three asaays by screening 103 cattle blood samples randomly collected from trypanosome endemic areas in western Kenya. The nested ITS based PCR, the single ITS based PCR and the species specific based PCR detected 28.1%, 26.2% and 10.7% of the samples respectively as positive for trypanosome infection. Nested ITS and single ITS PCRs' picked 3.8% and 1.9% as mixed infections respectively. Cohen kappa statistic used to compare agreements beyond chance between the assays showed highest degree of agreement (0.6) between the two ITS based tests, and the lowest (0.2) between the nested PCR test and the species specific PCR. The single ITS and nested ITS based diagnostic assays detected higher numbers of positive cases, and reduced the number of PCR reactions per sample to one and two respectively, compared to the five PCR reactions carried out using the species specific primers. This significantly reduced the labour, time and the cost of carrying out PCR tests, indicating the superiority of the ITS multi-species detection techniques. Reliable epidemiological studies are a prerequisite to designing effective tsetse and trypanosomiasis control programs. The present study established the suitability of using ITS based PCR assays for large-scale epidemiological studies.

## Findings

The development of good treatment and control strategies to protect livestock against trypanosomiasis requires accurate data regarding the disease epidemiology. This in turn depends on accurate diagnosis and definitive identification of causative trypanosome species. Most epidemiological studies have relied on parasitological methods for the demonstration of trypanosomes despite their limitations in terms of sensitivity and practicability [1]. Serological tests such as Ab-ELISA detection methods are not reliable for differenting current or post treatment infections [2]. Ag-ELISA assays have also been shown to be of insufficient sensitivity for any diagnostic value [3,4]. Accurate detection of trypanosomes in both the host blood and vectors now heavily depends on the highly sensitive and specific Polymerase Chain Reaction (PCR). Species specific primers amplifying pathogenic trypanosomes have been designed and used to characterise trypanosomes and in epidemiological studies [5-8]. However, pathogenic trypanosomes are known to occur and overlap in most of the tsetse infested belt [9]. As a result, screening of bovine blood samples from such endemic area may require upto six PCR reactions to test for each of the parasites; *Trypanosoma vivax, T. congolense *Savannah type, *T. congolense *Kilifi type, *T. congolense *Tsavo type, *T. congolense *Forest type and *T. brucei species*. This is time-consuming and costly, and requires advanced technical expertise. Attempts to combine already available primers into a single multiplex PCR have been discouraging due to lower sensitivity compared to individual species-specific PCR tests and the appearance of non-specific and non expected PCR products with some combinations of primers [10]. Recent researches now focus on multiple species identification using single primer sets based on ribosomal RNA gene sequences [10,11]. The internal transcribed spacer (ITS) region of ribosomal DNA presents the advantages of being a multi-copy locus (100–200 copies), having a small size (300–800 bp), and varying from one taxon to another but highly conserved in size in a given taxon, making it a preferred diagnostic target for a universal test [12-14]. Evaluation of ITS1 CF (forward primer) and ITS1 BR (reverse primer) for detecting all pathogenic trypanosomes showed good sensitivity levels for most species except T. *vivax*. To improve on the sensitivity of detecting *T. vivax*, designed primers for nested PCR targeting ITS1 and ITS2 were evaluated using field samples with reported success [15]. The aim of this study was to determine and compare the capacities of these three PCR assays to detect single and mixed trypanosome infections, and test their level of agreement using field samples obtained from the trypanosome endemic regions of western Kenya.

A total of 15 mls of blood were obtained through jugular venipuncture from each of the 103 randomly selected animals sampled from Teso and Suba regions of western Kenya. The blood samples were initially collected and maintained at room temperature in labelled falcon tubes containing 0.5 ml of 0.5 M EDTA and later frozen awaiting DNA extraction

**Figure 1:**
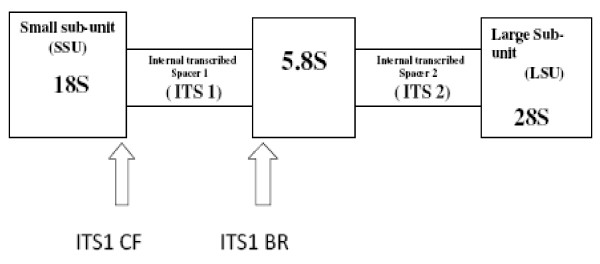
**Schematic diagram of rDNA showing ITS1 CF and ITS1 BR annealing positions**. The arrows show the annealing position for ITS1 CF, the 18S ribosomal small subunit, and ITS1 BR annealing at the 5.8S ribosomal sub-unit, to amplify the region ITS1, known to vary in size within trypanosome species. The large boxes (SSU and LSU) represent conserved coding regions, while the smaller boxes represent the spacer regions.

DNA was extracted from the frozen blood samples using the salt-out procedure described by Sambrook *et al*., (1989) with slight modifications [16]. All PCR amplifications were done in 25 μl reaction volumes containing final concentrations of 1 mM dNTP mix, 0.04 U/μl Go Taq^® ^DNA polymerase in 5× Go Taq^® ^Flexi PCR buffer, 1.5 mM MgCl_2 _(Promega), 100 ng/μl of each forward and backward primer for each trypanosome species using procedures described by Picozzi *et al*., [17]. All the 103 DNA samples were screened for the various trypanosome species namely; *T. congolense, T. vivax*, and *T. brucei *using species and type specific primers [18]. A set of purified genomic DNA from *T. congolense *savannah type, *T. congolense *kilifi type, *T. congolense *Forest type, *T. brucei *and *T. vivax *were included to serve as positive controls every time the screening was done. Table 1 shows the primer sequences, expected band sizes of PCR amplicons, and the PCR conditions used in screening for each trypanosome. These primers have been designed to amplify ITS1 region of rDNA which is known to vary in size within trypanosome species, except for members of Trypanozoon genus, and therefore differentiates trypanosomes by their ITS1 sizes [10,13,14]. The forward primer (ITS1 CF) anneals to the 18S while the reverse primer (ITS1 BR) anneals to the 5.8S region of rDNA amplifying ITS1 (Fig 1). PCR amplification using the primers ITS1 CF: 5' CCG GAA GTT CAC CGA TAT TG' 3 and ITS1 BR: 5' TTG CTG CGT TCT TCA ACG AA' 3. was performed on all the extracted DNA samples. Table 2 shows the trypanosome species screened and the expected band sizes of the PCR products. Increased sensitivity of ITS region amplification was achieved using nested PCR [15]. The outer primer sequences used in the nested PCR were ITS1 (5'-GAT TAC GTC CCT GCC ATT TG-3') and ITS2 (5'-TTG TTC GCT ATC GGT CTT CC-3'), and the inner primers sequences ITS3 (5'-GGA AGC AAA AGT CGT AAC AAG G-3') and ITS4 (5'-TGT TTT CTT TTC CTC CGC TG-3'). All primers were obtained from MWG Biotech (Germany). The expected product sizes obtained by calculating the distance between the primer locations as determined from the sequences for each trypanosome species present in bioinformatic databases and from amplification with these primers given in Table 3[15].

**Table 1:** Primer names, amplification conditions and the expected product sizes for each of the Species-specific primers.

Primer name	Primer sequence	Amplification conditions	Product size
TCS(F)ILO344	5'-CGA GAA CGG CAC TTT GCG A-3'	94°C for 3 min, 94°C for 30 sec, 60°C for 30 sec, 72°C for 30 sec, 72°C for 5 min (30 cycles)	316 base pairs
TCS(R)IL0345	5'-GGA CAA ACA AAT CCC GCA CA-3'		
TCK(F)ILO963	5'-GCG GCA GGT CGA CGG ATC-3'	94°C for 7 min, 94°C for 1 min, 55°C for 1 min, 72°C for 2 min, 72°C for 5 min (30 cycles)	294 base pairs
TCK(R)IL0968	5'-CCC TCG AGA ACG AGC A-3'		
TVW-1	5'-CTG AGT GCT CCA TGT GCC AC-3'	94°C for 3 min, 94°C for 30 sec, 60°C for 30 sec, 72°C for 30 sec, 72°C for 5 min (30 cycles)	150 base pairs
TVW-2	5'-CCA CCA GAA CAC CAA CCT GA-3'		
TBR-1	5'-CGA ATG AAT AAA CAA TGC GCA GT-3'	94°C for 3 min, 94°C for 1 min, 55°C for 1 min, 72°C for 1 min, 72°C for 5 min (30 cycles)	177 base pairs
TBR-2	5'-AGA ACC ATT TAT TAG CTT TGT GC-3'		

TCS – *Trypanosoma congolense *savannah subtype

TCK – *Trypanosoma congolense *kilifi subtype

TVW – *Trypanosoma vivax*

TBR – *Trypanosoma brucei*

**Table 2:** Trypanosome species and the expected band sizes on amplification with ITS 1 BR and ITS 1 CF primers

Trypanosome species	Approximate PCR amplification size product
Trypanozoon members (*T. brucei*)	480 bp
*T. congolense *Savannah	700 bp
*T. congolense *Kilifi	620 bp
*T. congolense *forest	700 bp
*T. vivax*	250 bp

**Table 3:** Trypanosome species and the expected band sizes on amplification with the nested ITS based primers (Cox *et al*, 2005)

Trypanosome species	Expected band size from NCBI database
Trypanozoon members (*T. brucei*)	1207 – 1224 bp
*T. congolense *Savannah	1413 bp
*T. congolense *Kilifi	1422 bp
*T. congolense *Forest	1513 bp
*T. vivax*	611 bp

McNemar's χ^2 ^and the kappa statistics (ĸ) were used to describe the level of agreements between tests using the single species specific primers (Test 1) and single ITS primers (Test 2), Test 1 and nested PCR (Test 3), Test 2 and Test 3, using SAS 9.1 statistical software. McNemar's χ^2 ^was carried out first to test whether there was test bias [19]. If the tests being compared are statistically significant, it suggests a serious disagreement between the tests whereas a non- significant test result indicates that the two proportions do not differ. The Cohen's kappa statistic was used to determine the diagnostic agreement between the three tests [20].

**Figure 2:**
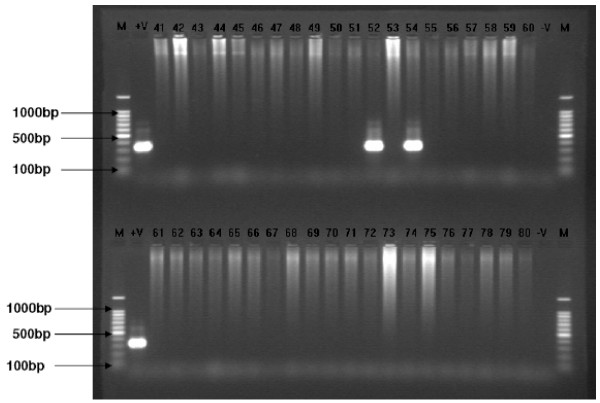
**Detection of *T. Congolense *savannah genomic DNA amplified with TCS 1 and 2 primers in field samples**. Representative gel image of electrophoresis of reference DNA samples (+v), test samples labelled 41 – 80, negative controls (-v) and 100 base pair ladders labelled 'M'. The reference DNA has been amplified with a product size of 316 bp, samples number 52 and 54 being positive, and no bands for the negative controls.

Out of the 103 cattle blood samples screened for trypanosome infections using the species specific primers, 10.7% tested positive. Two *T. congolense *genotypes; savannah and Kilifi sub-types were identified at frequency of 2.9% and 0.97% respectively. 3.9% of the samples tested positive for *T. vivax*, and 2.9% for *T. brucei*. PCR using species specific primers did not pick any mixed infection from any of the samples. Figure 2 shows a gel image of DNA samples screened for *T. congolense *savannah and *kilifi *subtypes, *T. brucei *and *T. vivax *respectively.

**Figure 3:**
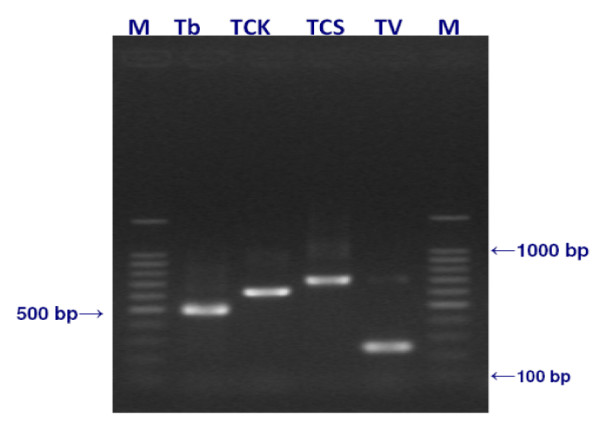
**Gel electrophoresis of Trypanosome control DNA amplified with the ITS1 CF & BR primers**. *T. brucei *(T.b) giving a product of approximately 480 bp, *T. congolense *Kilifi (TCK) approximately 620 bp, *T. congolense *savannah (TCS) approximately 700 bp, and *T. vivax *approximately 250 bp.

**Figure 4:**
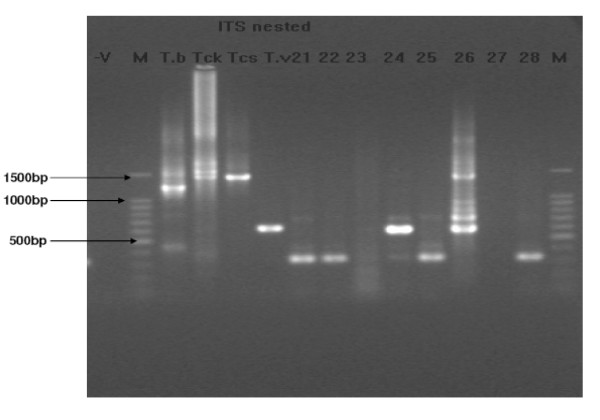
**Gel electrophoresis of Trypanosomes using ITS nested primers**. Expected band sizes are 1207–1224 bp for *T. brucei *(T. b), 1422 bp for *T. congolense *kilifi (Tck) and 1413 bp savannah (Tcs)sub-types, and 611 bp for *T. vivax *(T. v) DNA samples. Test samples are labelled 21–28, negative control (-v) and 100 base pair ladders labelled 'M'. Sample numbered 24 & 26 are positive for *T. vivax*, with number 26 being a mixed infection with *T. congolense *savannah. No amplification on the negative controls.

The results of PCR using ITS1 BR and ITS1 CF primers which amplifies the ITS 1 region identified 26.2% (27 animals, n = 103) as positive for trypanosome infection. *T. vivax *was the most frequently detected (17.5%) followed by *T. congolense *and *T. brucei *at 4.9% and 3.9% respectively. Two individuals (1.9%) were detected with mixed infections of *T. vivax *and *T. brucei*. Figure 3 shows the gel image of amplicons obtained using these ITS 1 primers. Nested PCR using ITS 1 and 2 outer primers, and ITS 3 & 4 inner primers detected a total of 29 animals (28.1%) positive for trypanosomes, with 3.8% as mixed infections of *T. vivax *and *T. congolense. T. vivax *was most frequently detected (23.3%) followed by *T. congolense *and *T. brucei *at 6.7% and 1.9% respectively. Figure 4 is a gel image showing samples screened using the nested PCR method. A descriptive comparison among the three PCR tests showed frequency of positive results among the 103 samples to be 28.1% (29/103), 26.2% (27/103), and 10.7% (11/103) for nested PCR ITS 1 & 2 assay, Single ITS1 PCR, and Species specific primers PCR, respectively. McNemar's test results were highly significant between the species specific test and the single ITS1 based tests (χ^2 ^= 10.7, df = 1, *p *= 0.001), and between Species specific test and the nested ITS 1 & 2 test (χ^2 ^= 14.44, df = 1, *p *= 0.0001) indicating a disagreement between the tests. McNemar's test result for the two ITS based assays was not significant (χ^2 ^= 1.0, df = 1, *p *= 0.3173) indicating agreement between them. Calculations of the Cohen's kappa gave results ranging from slight agreement (0.2) between the species specific and Single ITS test, to reasonable agreement (0.6) between the two ITS based tests.

Trypanosomiasis in the field will present both in the chronic and acute forms depending on whether it is in the endemic or epidemic epidemiological form. Whereas parasitological methods are reported as having an almost equal sensitivity to PCR in detecting infections in the acute phase, they exhibit very low sensitivity in the chronic phase characterized by low parasitaemia, in which PCR will be two to three times more sensitive [21]. There have been debates on the relative merits of microscopy and PCR methods for the detection of trypanosome infection but this should focus on advantages of each method for specific purposes. For instance, field studies comparing the use of PCR and microscopic observation of the Buffy coat have reported higher sensitivity with PCR, which should be important in epidemiological studies but not necessarily for practical clinical diagnosis to determine treatment regimes [22,23].

The diagnostic performance variables such as sensitivity and specificity for each of the assays used in this study have been estimated in separate studies describing them [6,14,15]. However besides estimating diagnostic performance variables of available PCR tests, assessing the tests for agreement is important for practical applications of the different PCR diagnostic assays. The choice of the method to use for each study should consider the cost implications as well as the need for accuracy.

The two ITS based assays utilizing the same diagnostic target showed a higher diagnostic capacity compared to the species specific tests. The difference was mainly due to the high numbers of *T. vivax *picked by the ITS based assays. Accurate diagnosis of *T. vivax *species is important since it is the etiological cause of the severe hemorrhagic disease in cattle. This is made even more important from the fact that *T. vivax *is also transmitted mechanically. This means that whereas tsetse control would bring the prevalence other pathogenic trypanosomes down, *T. vivax *would possibly persist. Diagnosing *T. vivax *therefore, would spell the need for other trypanosomiasis control strategies in cattle such as chemotherapy besides tsetse control.

Although the single PCR ITS based diagnostic test is reported to have a low sensitivity against *T. vivax*, our results indicated it was able to detect much more (17.5%) positives for *T. vivax*, compared to the TVW primers (3.9%) [10,14]. These results support other studies suggesting that TVW primers target certain DNA sequences that are not conserved in all *T. vivax *isolates, resulting to false-negatives [22,23]. In addition, low sensitivity could be due to the TVW primers targeting molecules that are low in copy numbers as compared to ITS-PCR whose target gene could be higher in copy numbers [24,25]. Results from this study confirm the superiority of the nested PCR method which was developed to improve the sensitivity observed with the single ITS test in detecting *T. vivax *infections.

The three tests did not differ significantly in detecting *T. brucei *and picked almost equal numbers of *T. brucei*. The TBR 1 and TBR 2 primers target a 177 bp repeat sequence which occurs in a high copy number of approximately 1000 copies, which explains the comparable sensitivity of these primers with the ITS based assays. A statistical analysis of the test agreements indicates a significant difference in the diagnostic capacity between the species-specific primers tests and the other two universal tests largely due to *T. vivax *numbers. Between the two universal tests, the McNemar's test for bias was not significant and the kappa value indicated reasonable agreement, implying that the two tests presumably detect the parasites equally well.

Prohibitive costs and widespread perception that diagnostic PCR technology is complex slows down its adoption. For Instance in this study, while using the species-specific diagnostic method, five different PCR assays per sample were required to screen for *T. vivax, T. brucei *and the three subtypes of *T. congolense*; Savannah, Kilifi and Tsavo. The tests consumed more time and labour, and a higher cost compared to the two ITS based tests. If the cost constraints are overcome, efforts should be directed towards making diagnostic PCR technology automated, minimizing sample handling and decreasing the possibility of contamination, while raising the potential to function efficiently in the hands of moderately trained technical staff [1].

The use of ITS based primers as a universal diagnostic test for all pathogenic trypanosomes considerably overcomes the above constraints. By reducing the number of reactions per sample to one or two, the tests effectively reduces the cost of PCR by two to three times [14] while reducing the time required for diagnosis. The nested PCR gives the advantage of detecting the highest number of trypanosome infections but requires two reactions slightly increasing the cost and time compared to the single ITS based test. [15] conservately estimates a reduction to cost and time by a factor of four while using the nested PCR.

Although microscopy remains the most appropriate method for the clinical diagnosis in a field setting, it lacks sensitivity to be considered as a gold standard and the molecular methods provide an alternative gold standard for the detection of trypanosomiasis. The ITS based assays definitely make PCR diagnosis more accurate, faster and less costly to carry out for large numbers of samples. This promises the possibility of carrying out large-epidemiological studies on African trypanosomiasis in a simple and cost-effective way.

## Competing interests

The authors declare that they have no competing interests.

## Authors' contributions

JOJ, ROM, and SMT conceived the study. SMT, JOJ, and FAM carried out essential molecular analyses. FAM contributed expertise in Trypanosome diagnostic design. JOJ and SMT drafted the manuscript, and all the authors contributed significantly to editing the manuscript. All authors read and approved the final manuscript.
